# Gas Chromatography Time-Of-Flight Mass Spectrometry (GC-TOF-MS)-Based Metabolomics for Comparison of Caffeinated and Decaffeinated Coffee and Its Implications for Alzheimer’s Disease

**DOI:** 10.1371/journal.pone.0104621

**Published:** 2014-08-06

**Authors:** Kai Lun Chang, Paul C. Ho

**Affiliations:** Department of Pharmacy, Faculty of Science, National University of Singapore, Singapore, Singapore; Swinburne University of Technology, Australia

## Abstract

Findings from epidemiology, preclinical and clinical studies indicate that consumption of coffee could have beneficial effects against dementia and Alzheimer’s disease (AD). The benefits appear to come from caffeinated coffee, but not decaffeinated coffee or pure caffeine itself. Therefore, the objective of this study was to use metabolomics approach to delineate the discriminant metabolites between caffeinated and decaffeinated coffee, which could have contributed to the observed therapeutic benefits. Gas chromatography time-of-flight mass spectrometry (GC-TOF-MS)-based metabolomics approach was employed to characterize the metabolic differences between caffeinated and decaffeinated coffee. Orthogonal partial least squares discriminant analysis (OPLS-DA) showed distinct separation between the two types of coffee (cumulative Q^2^ = 0.998). A total of 69 discriminant metabolites were identified based on the OPLS-DA model, with 37 and 32 metabolites detected to be higher in caffeinated and decaffeinated coffee, respectively. These metabolites include several benzoate and cinnamate-derived phenolic compounds, organic acids, sugar, fatty acids, and amino acids. Our study successfully established GC-TOF-MS based metabolomics approach as a highly robust tool in discriminant analysis between caffeinated and decaffeinated coffee samples. Discriminant metabolites identified in this study are biologically relevant and provide valuable insights into therapeutic research of coffee against AD. Our data also hint at possible involvement of gut microbial metabolism to enhance therapeutic potential of coffee components, which represents an interesting area for future research.

## Introduction

There is a growing body of epidemiological evidence that supports therapeutic roles of coffee consumption against development of Alzheimer’s disease (AD). In a study with follow-up of 21 years, people who drank 3–5 cups of coffee per day during midlife were observed to have 65% reduction in risk of developing AD later in life as compared to those who drank little or no coffee [1]. A meta-analysis of pooled epidemiological studies followed shortly, reporting protective effects of coffee consumption against AD, but the methodological heterogeneity imposes limitations on interpretation of the findings [2].

Recognizing that epidemiologic studies are not direct evidence, Cao and colleagues obtained the first direct human evidence to support benefits of coffee consumption in AD [3]. They observed that mild cognitive impairment (MCI) patients with higher plasma caffeine levels have delayed onset or lower risk of dementia during a 2–4 year follow-up period, and most of caffeine sources for study subjects were traced back to coffee consumption [3]. This study strengthened therapeutic roles of coffee consumption in preventing AD, and proposed coffee consumption as prophylactic intervention far before surfacing of AD symptoms.

Interestingly, some recent studies revealed that therapeutic benefits of coffee were not due to caffeine alone. One study showed that improvements of cognition and psychomotor behaviours in aged rats were due to coffee, and not caffeine itself [4]. In line with these observations, crude caffeine – a by-product of coffee decaffeination process – was observed in another study to have greater therapeutic effect on memory impairment in AD mouse model than pure caffeine [5]. Specifically, the study reported that administration of crude caffeine, but not pure caffeine, reduced amyloid burden, improved antioxidant activity and enhanced glucose uptake in AD mouse model [5].

Decaffeinated coffee, on the other hand, has had mixed results when it was investigated for its therapeutic potential in AD. It had been shown to improve insulin resistance and brain energy metabolism in mice [6]. However, acute administration of decaffeinated coffee was observed to have very limited beneficiary effect on glucose homeostasis, a metabolic process closely implicated in both type 2 diabetes and AD [7]. More recently, researchers started to systematically compare both caffeinated and decaffeinated coffee for their therapeutic effects in AD. One study reported potentiating effect of other unknown coffee components on caffeine’s benefits against AD, therefore making caffeinated coffee the therapeutically superior [8]. In another study, consumption of caffeinated coffee, but not decaffeinated coffee or pure caffeine, was observed to be therapeutic against oxidative stress, a pathological marker common to AD [9].

To produce decaffeinated coffee, coffee beans could be subjected to several different decaffeination processes, namely solvent-based, water-based, and supercritical carbon dioxide-based decaffeination methods [10]. These processes differ in costs and types of solvents used to extract caffeine, but one thing in common for all these different methods is that their decaffeination process removes more than just caffeine. Taking water-based decaffeination process for example, it is known that its extraction step results in loss of water-soluble components of coffee beans which are in excess of 20% by weight; therefore the extraction water is supplemented with non-caffeine soluble solids to minimize loss of these water-soluble components through extraction [10]. Supercritical carbon dioxide-based decaffeination method, a process despite often touted for its high extraction selectivity for caffeine, removes more than just caffeine, resulting in the brown colour of crude caffeine [11]. Not surprisingly, it has been shown that the crude caffeine contains a variety of non-caffeine bioactive phytochemicals [11], and as previously mentioned, crude caffeine exerted therapeutic effects on AD mouse model which were not observed with pure caffeine treatment [5]. Perhaps the most immediate sentiment of decaffeinated coffee would be the change in aromas and flavours as compared to caffeinated coffee, clearly suggesting that significant portions of non-caffeine phytochemicals have been stripped away from the coffee beans by decaffeination processes.

Although decaffeinated coffee has been available in the market for a long time, there are limited data on characterization of chemical composition profiles that differentiate caffeinated and decaffeinated coffee. One study investigated alterations in chlorogenate levels in coffee following water-based decaffeination process, as chlorogenate was perceived to be one of the major phytochemicals in coffee that is associated with health benefits [12]. However the evidence for beneficial effects of chlorogenate was inconclusive and one study reported no beneficial effect on glucose metabolism was observed when chlorogenate was given to human patients at risk for type 2 diabetes [7]. Some studies were set out to investigate alterations in phenolic contents of coffee following decaffeination process, but contradictory data had been reported [13], [14]. These represent a significant research gap in the current literature between evidence of coffee’s beneficial health effects with chemical composition of its bioactive components. Bridging this gap is especially important with the emergence of aforementioned reports on therapeutic differences of caffeinated and decaffeinated coffee for prevention or treatment of AD.

Metabolic profiling approach presents itself as a suitable tool for characterization of chemical composition in coffee samples. This approach systematically profiles all small molecules present in samples and utilizes data mining tool to sieve out meaningful data by comparing metabolic profiles of caffeinated and decaffeinated coffee. A few recent studies applied such metabolomics approach on coffee samples to determine coffee origins [15], [16], as well as to authenticate prized coffee product [17]. However, none of these studies used the metabolomics approach to systematically characterize and understand chemical compositions of coffee, and applied it within a drug discovery setting. This study aimed to employ metabolomics tool to study the chemical differences in coffee rendered by decaffeination process, and hoped to identify a list of discriminant compounds that could have contributed to the therapeutic superiority of caffeinated over decaffeinated coffee for AD treatment.

With the availability of a variety of high-throughput analytical instruments, metabolomics approach has to be appropriately matched with the right choice of analytical platform to achieve study’s aims more efficiently. GC-MS has been used widely in metabolomics studies due to its excellent sensitivity and the availability of large commercial electron ionization (EI) spectral libraries, which was made possible by highly robust and reproducible EI mass spectra. As a result, highly efficient and straightforward identification of metabolic peaks is a strong advantage for GC-MS-based metabolomics approach, especially for a non-targeted approach. This study proposed to employ GC-TOF-MS as the platform for metabolomics of both caffeinated and decaffeinated coffee. A recent report by Jumhawan et al. employed GC-quadrupole-MS for metabolomics of coffee samples [17]. However, a quadrupole MS loses its sensitivity when operated in scanning mode due to compromised duty cycle, and our TOF-MS could be more superior to a quadrupole-MS when employed in a non-targeted metabolomics setting. Findings from our study were compared against Jumhawan et al’s study under Results and Discussion.

This is the first time metabolomics was used as a tool to profile the chemical differences between caffeinated and decaffeinated coffee. Our study successfully demonstrated the viability of GC-TOF-MS as an analytical platform for metabolomics analysis of coffee samples. On top of that, metabolic profiling approach was found to be an effective method in elucidating chemical differences between caffeinated and decaffeinated coffee. Novel findings reported in this study could shed light on optimization of decaffeination processes and therapeutic research on coffee consumption for prevention or treatment of AD.

## Materials and Methods

### Chemicals and reagents

2% Methoxamine hydrochloride in pyridine (MOX reagent) and N-methyl-N-(trimethylsilyl) trifluoroacetamide (MSTFA) with 1% trimethylchlorosilane (TCMS) were purchased from Thermo Fisher Scientific (Waltham, MA). All other reagents used were of analytical grades.

### Caffeinated and decaffeinated coffee samples

NESCAFÉ GOLD caffeinated and decaffeinated coffees (Nestlé, Singapore) were purchased commercially. Both coffee options were available as freeze-dried granules, and water-based decaffeination method was used by the manufacturer. For both caffeinated and decaffeinated coffee, 400 mg of freeze-dried granules were transferred into a clean 50-ml falcon tube, and 40 ml of Milli-Q water (warmed to 80°C) was added to make a coffee solution. The solution was vortex-mixed to ensure complete dissolution of the freeze-dried granules. The respective coffee solution was prepared in 5 replicates for both caffeinated and decaffeinated coffee. Freshly prepared coffee solutions were immediately subjected to sample preparation step for subsequent metabolomics analysis.

### Sample preparation for metabolomics analysis

200 µl of each coffee sample was transferred to clean 2-ml centrifuge tube and 1.0 ml of chilled methanol was added to each tube. All mixtures were vortex-mixed at high speed for 5 minutes, followed by centrifugation (14,000 g) for 20 minutes at 4°C. 950 µL of supernatant from each sample was then carefully transferred into clean, pre-silanized glass tubes and evaporated to dryness at 50°C under a gentle stream of nitrogen gas using TurboVap nitrogen evaporator (Caliper Life Science, Hopkinton, MA, USA). 100 µL of anhydrous toluene (stored with sodium sulfate) was added to each dry residue, vortex-mixed for 1 minute, and dried again at 50°C under nitrogen gas. This was to ensure complete elimination of water which might interfere with the subsequent sample preparation steps. Then, 50 µL of MOX reagent was added to each dried extract, vortex-mixed for 2 minutes and incubated at 60°C for 2 hours as a methoximation step. Derivatization reaction aimed to increase the volatility of polar metabolites was then initiated by adding 100 µL of MSTFA (with 1% TMCS) to each sample, vortex-mixed for 2 minutes, and incubated at 60°C for 1 hour. Following the incubation, each sample was vortex-mixed for 2 minutes and carefully transferred to the GC autosampler vials for subsequent GC-TOF-MS analysis.

### GC-TOF-MS data acquisition and preprocessing

Agilent 7890A Gas Chromatography (Agilent Technologies, Santa Clara, CA) coupled to PEGASUS 4D Time-of-Flight Mass Spectrometer (LecoCorp., St. Joseph, MI) was used for separation and detection in our GC-TOF-MS setup. Column used was a DB-1 GC column (Agilent Technologies) with a length of 22.9 m, internal diameter of 250 µm, and film thickness of 0.25 µm. Carrier gas used was helium at a flow rate of 1.5 ml/min. Split ratio for injector was set to 1∶10, with a total injection volume of 1 µL. Front inlet and ion source temperatures were both kept at 250°C. Oven temperature was set to equilibrate at 70°C for 0.5 minute, before initiation of sample injection and GC run. Following sample injection, oven temperature was maintained at 70°C for another 0.2 minute, before it was increased at a rate of 8°C/min to 270°C, and held at 270°C for 5 minutes. Temperature was then further increased by 40°C/min to reach 310°C and held for another 5 minutes to complete the run. The MS detection was operated in EI mode (70 eV) with detector voltage of 1800 V. Full scan mode with mass range of m/z 50–600 and acquisition rate of 15 Hz was used as data acquisition method. Acquisition delay was set at 195 seconds to avoid detection of by-products from sample derivatization step. Chromatogram data acquisition, baseline correction, peak deconvolution, analyte alignment, peak area integration, and analyte identification by mass spectral searches (based on National Institute of Standards and Technology and Fiehn Rtx5 libraries) were performed using the LECO ChromaTOF software version 4.21. Peaks with similarity index of 70% or more were assigned putative metabolite identities based on the mass spectral libraries. Similarity index of 70% or more was chosen because this cut-off value afforded 100% accuracy in analyte identification based on our previous experience (confirmed by co-injection of commercial standards) [18]. Baseline offset, minimum peak width, signal to noise ratio and number of apexing masses were set at 0.5, 2.5 s, 100, and 3, respectively. Unique mass from each detected metabolite was used to calculate the integration area of each metabolite peak. Peak table was generated using Calibration feature of ChromaTOF software, with similarity threshold set at 70%. To ensure consistency in GC-TOF-MS data acquisition, we included quality control (QC) analysis, and maximum acceptable CV of 20% was set as the cut-off value for inclusion of metabolic peaks in subsequent analyses. Caffeinated and decaffeinated coffee each served as its own QC samples. Both caffeinated and decaffeinated coffee samples were injected in an interspersed manner to minimize introduction of procedural artefacts and ensure good data reliability in our QC and subsequent analyses. Metabolites that were not assigned a putative metabolite identity (similarity index <70%) or had CV higher than 20% in QC analysis were excluded from subsequent analyses. Resulting metabolic data was processed using total integral area normalization method, where area of each included peak in one sample was divided by the sum of all included peaks in the same sample.

### Multivariate data analysis

The normalized data were mean-centered and unit-variance scaled before being subjected to principal component analysis (PCA) (SIMCA-P software version 13.0, Umetrics, Umeå, Sweden). PCA was used to observe clustering trends, as well as to identify and exclude outliers in the data. After an initial surveillance of data using PCA, they were subjected to partial least squares discriminant analysis (PLS-DA) to build a discriminant model. Model validity and potential over-fitting of PLS-DA model were checked by performing 500 permutation tests and visualized using a validation plot. After PLS-DA model passed model validation, the same data were subjected to orthogonal partial least squares discriminant analysis (OPLS-DA) for better identification and interpretation of discriminant metabolites that are responsible for differentiation between caffeinated and decaffeinated coffee samples. To generate a list of potential discriminant metabolites, variable importance plot (VIP) cut-off value was set at 1.00. Two-tailed independent t-test with Welch’s correction was then used to compare means of these potential discriminant metabolites between the two coffee groups and Bonferroni-adjusted *P*-value was used to determine significance. Discriminant metabolites that have both VIP ≥1.00 and *P*-value lower than Bonferroni-adjusted significance levels were identified as discriminant metabolites between caffeinated and decaffeinated coffee. Biologically relevant information regarding the identified discriminant metabolites were sought from past literature for interpretation and discussion of our findings.

## Results and Discussion

### GC-TOF-MS was established as a suitable platform for metabolomics analysis of coffee samples

**Figure 1:**
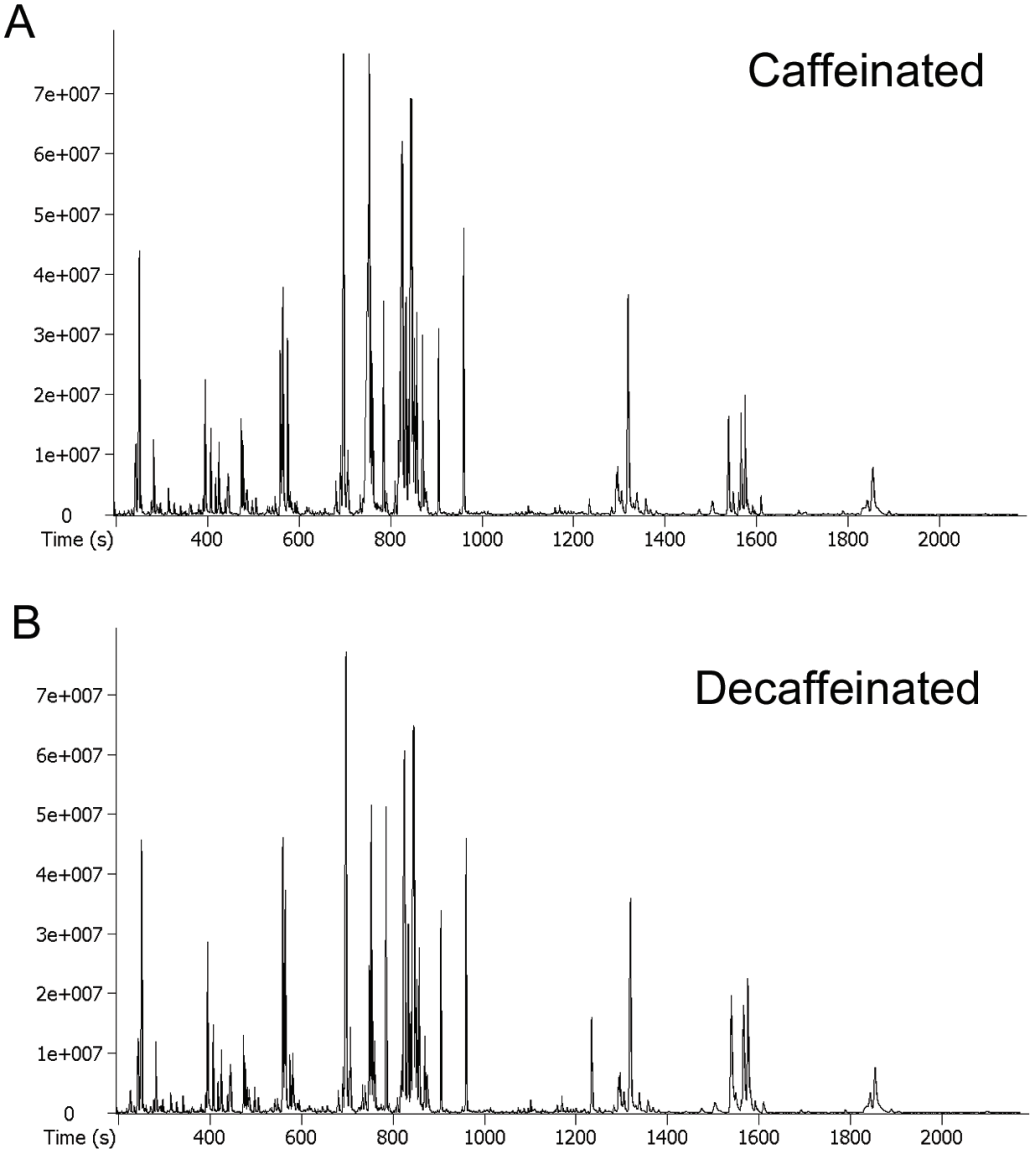
Metabolomic profiling of coffee samples using GC-TOF-MS. Panel A shows the representative GC-TOF-MS chromatogram of caffeinated coffee sample, and panel B shows the representative GC-TOF-MS chromatogram of decaffeinated coffee sample.

We first established suitability of GC-TOF-MS as an analytical platform for metabolomics analysis of coffee samples by carrying out QC analysis. 5 different samples were made for each of the caffeinated and decaffeinated coffee to be used as QC samples. Total ion chromatograms from GC-TOF-MS analyses of injected samples for both caffeinated and decaffeinated coffee were shown in **Figure 1A and 1B**, respectively. Both chromatograms displayed extensive regions of overlapping, suggesting that both coffee products were derived from similar source or batch of coffee beans. In total, 332 metabolic peaks were detected in both caffeinated and decaffeinated coffee samples. Out of these 332 metabolites, 97 distinct metabolites were assigned putative metabolite identities based on our pre-defined matching criteria (similarity index≥70%) against mass spectral libraries. QC analysis was performed on these 97 identified metabolites, and it was observed that none of them had CV more than 15% in both caffeinated and decaffeinated coffee groups. With the exception of 2-deoxy-D-galactose (CV = 11.5%, QC for caffeinated coffee) and L-proline (CV = 10.8%, QC for decaffeinated coffee), all other metabolites had CV of 10% or lower, indicating presence of minimal variations in our data acquisition. Therefore, our study successfully established GC-TOF-MS as a robust and highly reproducible platform for metabolomics analysis of coffee samples.

On top of good reproducibility, our analytical platform also demonstrated high sensitivity when compared against GC-quadrupole-MS used in a recent metabolomics study on coffee samples [17]. In their attempt to perform non-targeted metabolomics as a novel method to authenticate the highly prized Civet coffee, Jumhawan et al. detected a total of 182 metabolic peaks and identified 26 of them by matching against mass spectral libraries [17]. In comparison, we detected 332 metabolic peaks and successfully assigned 97 putative metabolite identities by library matching, despite our usage of a lower MS scan speed (15 Hz) as compared to scan speed used in Jumhawan’s study (10000 Hz). This could be due to compromised duty cycle when quadrupole MS is operated in scanning mode. In addition, our longer GC run time might offer better separation of peaks, hence offering better peak resolution during analysis.

### Multivariate data analysis for metabolomics data

**Figure 2:**
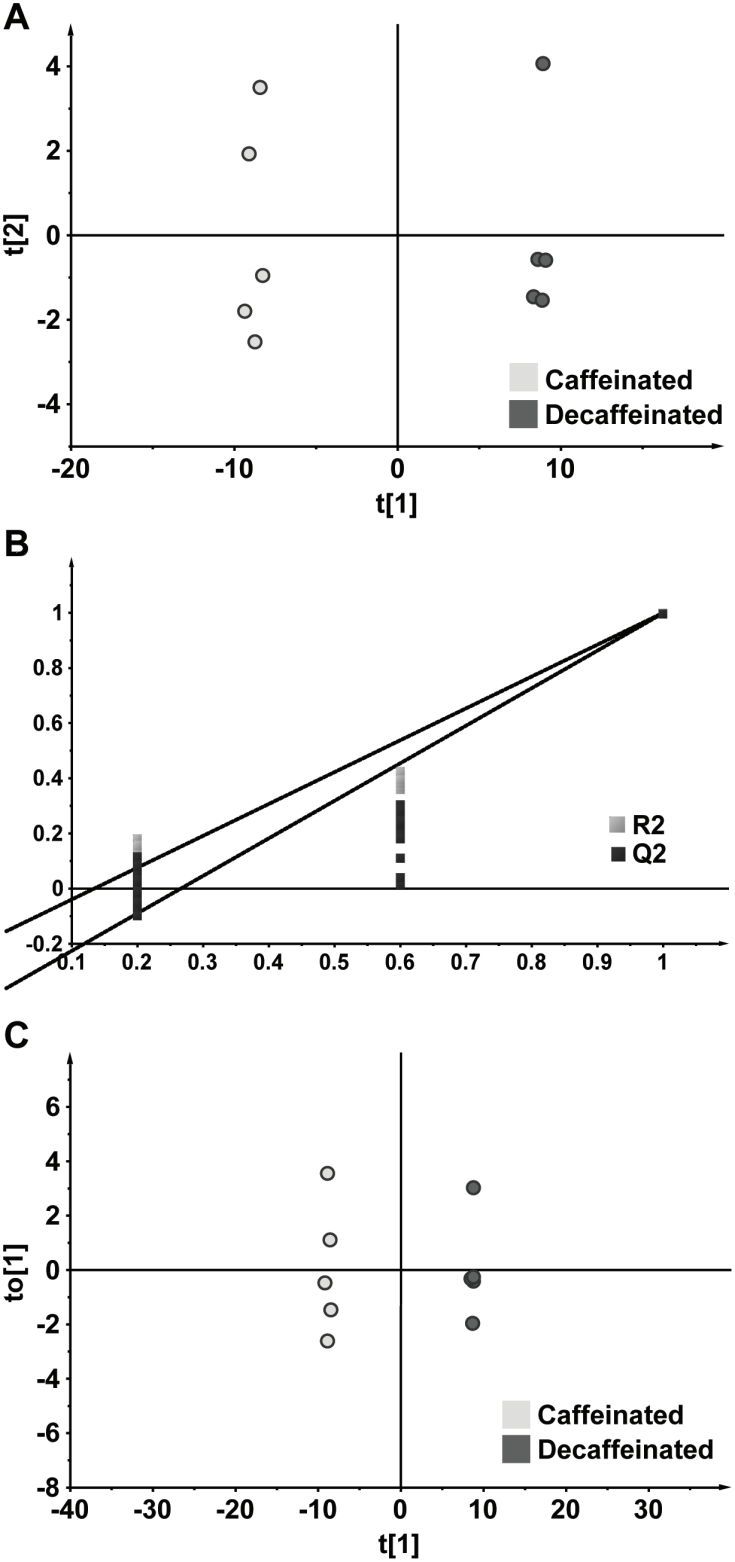
Multivariate data analysis of metabolites in the caffeinated and decaffeinated coffee samples. Panel A shows the PCA scores plot for caffeinated and decaffeinated coffee samples, panel B displays the model validation plot for PLS-DA model between caffeinated and decaffeinated coffee samples, and panel C illustrates the OPLS-DA model between caffeinated and decaffeinated coffee samples (1 predictive component, 1 orthogonal component, R^2^(Y) = 1, Q^2^(cum) = 0.998).

After we had established suitability of GC-TOF-MS for metabolomics analysis of coffee samples, we proceeded to carry out multivariate data analysis on metabolic data for both caffeinated and decaffeinated. PCA of both caffeinated and decaffeinated coffee samples displayed distinct clustering trend on the scores plot, suggesting that their metabolic profiles differ extensively from each other (**Figure 2A**). None of the samples from both groups fall outside the Hotelling’s T2 tolerance ellipse which denotes 95% confidence limit of the model, indicating that no outlier was present among the samples analysed. A PLS-DA model between caffeinated and decaffeinated coffee was generated, and validation plot for PLS-DA model indicated clearly that no over-fitting was observed and the model is valid as all Q^2^ values calculated for permuted datasets were lower than actual Q^2^ value and the regression line of Q^2^ values intersected y-axis below zero (**Figure 2B**). OPLS-DA model constructed using the same data is presented in **Figure 2C** (1 predictive component, 1 orthogonal component, R^2^(Y) and Q^2^(cum) were 1 and 0.998, respectively). R^2^(Y) is the fraction of the sum of squares of all Y-values explained by the current latent variables, and Q^2^(cum) is the cumulative Q^2^ for the extracted latent variables. Q^2^ is defined by the following equation:
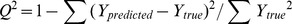



The high value of Q^2^(cum) for the OPLS-DA model indicates that separation between metabolic profiles of the caffeinated and decaffeinated coffee is strong and highly robust. Predictive component modelled 84.4% of variation among the 97 identified metabolites, while orthogonal component modelled only 4.0% of the variation. This shows that most of the variations in metabolic data could be explained by the separation between caffeinated and decaffeinated coffee. Therefore, the OPLS-DA model built using metabolic data from both coffee groups was shown to be valid in our analyses and subsequently used for discovery of discriminant metabolites.

### List of discriminant metabolites that differentiate caffeinated from decaffeinated coffee

A total of 97 putatively identified metabolites were used to build the OPLS-DA model as discussed under section 3.2. Based on the OPLS-DA model, a list of 69 potential discriminant metabolites (VIP ≥1.00) that were responsible for separation in the OPLS-DA model was generated. All of these 69 metabolites achieved significance (P<0.0007, Bonferroni-adjusted significance level) when their means were compared using two-tailed independent t-tests with Welch’s correction, confirming their significant contributions as discriminant metabolites to separation between caffeinated and decaffeinated coffee. A summary of the 69 discriminant metabolites can be found in **Table S1**.

In total, 37 compounds were detected to be higher in caffeinated coffee. These include several phenolic compounds which are benzoate and cinnamate derivatives. In particular, benzoate itself was 21% higher in caffeinated coffee, while two other monohydroxybenzoates, namely 3-hydroxybenzoate and 4-hydroxybenzoate were 151% and 33% higher in caffeinated coffee, respectively. On the other hand, two dihydroxybenzoates, namely gentisate and protocatechuate were 20% and 11% higher in decaffeinated coffee. Interestingly, dihydroxybenzoates were reported to possess higher antioxidative capacities than monohydroxybenzoates [19], [20]. However, our findings clearly showed that only monohydroxybenzoates were higher in caffeinated coffee; whereas dihydroxybenzoates were higher in decaffeinated coffee instead. Coupled with previous reports of therapeutic superiority of caffeinated over decaffeinated coffee for prevention of AD, our study suggests that therapeutic advantages of caffeinated coffee could be due to presence of markedly higher levels of monohydroxybenzoates, which are metabolized via microbial degradation into other phenolic compounds and constituents of citrate cycle [21]. This is especially an important factor when tens of trillions of gut microbes serve as connector between diet and health in human [22]. Caffeate (a cinnamate derivative) was also detected in our study, but no difference in caffeate was detected between the two coffee groups. Even though cinnamate-derived compounds were reported to be more efficient antioxidants than benzoate-derived ones [19], our data suggest that caffeate did not contribute to therapeutic superiority of caffeinated coffee. This finding is in agreement with previous report, which showed that chlorogenate (another cinnamate derivative) has no beneficial effect on glucose metabolism in patients at risk for type 2 diabetes [7]. Our findings suggest that antioxidative capacities of benzoic and cinnamate-derived phenolic compounds could be subjected to modifications by extensive gut microbial metabolism upon consumption. Further studies are needed to uncover these missing links, which could potentially have a significant impact on how we perceive the connection between coffee consumption and health outcome.

Some organic acids were shown to be present at higher levels in caffeinated coffee. These include L-2-hydroxyglutarate (+91%), erythronate (+67%), methylsuccinate (+61%), and succinate (+20%). All these compounds were reported to be decreasing in urinary concentrations with increasing age in a previous study [23]. Although the age of subjects employed in that particular study was only up to 12 years old, their findings indicated a clear decreasing trends in urinary excretion of these compounds with increasing age in their subjects, demonstrating the relevance of these compounds to developmental health. Our study showed that these compounds were all higher in caffeinated coffee, but whether is there beneficial dietary effects if consumed over the long term remained yet to be defined. 5-hydroxyvalerate is another organic acid which was also observed to be higher in caffeinated coffee (+138%). This compound was previously reported as an antioxidant present in citrus peel [24], and its higher level in caffeinated coffee could contribute to higher antioxidative activity and free radical scavenging capacity, therefore conferring caffeinated coffee its therapeutic superiority over decaffeinated coffee.

Other compounds that were observed to be higher in caffeinated coffee could also give caffeinated coffee a therapeutic advantage over decaffeinated option for prevention of AD. Fumarate, a fatty acid which is also a component of citrate cycle, was found to be higher in caffeinated coffee (+195%). Interestingly, fumarate and its esters had been investigated for their neuroprotective and antioxidative effects, which was believed to be due to its salvaging effect on perturbed citrate cycle [25]. Shikimate was detected to be 60% higher in caffeinated coffee than decaffeinated one. This compound is widely present in edible plants, and its production in plant is increased as an antioxidative response to wounding stress [26]. It had also been shown that shikimate can be metabolized by gut microbes to cyclohexanecarboxylate, which will then be aromatized in mammalian tissues (Wheeler 1979).

Besides antioxidants, other detected compounds could also offer therapeutic advantages to caffeinated coffee via different mechanisms. L-rhamnose, a deoxy sugar that has been shown to exert inhibitory effects on lipogenesis upon consumption [27], was observed to be higher in caffeinated coffee (+59%). 2-Furoate, which was reported to possess lipid lowering effects in a previous study [28], was also higher in caffeinated coffee (+20%). Dysregulation of lipid metabolism has been closely associated with development of AD, and could serve as a potential therapeutic target for AD treatment [29]. Our findings suggest that L-rhamnose and 2-furoate could act as lipid metabolism regulators, hence making caffeinated coffee the more desirable option for prevention of AD. Another potential therapeutic mechanism could be coming from the higher picolinate in caffeinated coffee (+31%). It had already been shown that dietary picolinate enhances absorption of dietary zinc in human [30], and zinc deficiency had been previously discussed as a factor for AD pathogenesis [31]. Our data showed higher levels of picolinate in caffeinated coffee as compared to decaffeinated counterpart, and this could potentially enhance zinc absorption over long-term consumption and contribute to prevention of AD.

Contrary to our original expectations, decaffeination process did more than stripping components away from coffee beans. Several compounds were actually detected at higher levels in decaffeinated coffee, suggesting that the decaffeination process could have enhanced their levels. These compounds include pyruvate (+785%), 2-ketobutyrate (+309%), and malate (+96%), which are closely associated with energy metabolism. Several amino acids were also detected at higher levels in decaffeinated coffee, namely L-aspartate (+129%), L-proline (+85%), L-phenylalanine (+76%), L-alanine (+59%), L-valine (+54%), and glycine (+53%). Interestingly, we detected higher levels of trigonelline in decaffeinated coffee (+41%). Trigonelline, a vitamin B3 precursor, is often described as a major component of coffee that exerts beneficial effect on glucose metabolism, a trait closely associated with development of AD [32]. However, contradictory evidence showed that it had limited anti-diabetic effects in human patients [7], therefore implying its limited therapeutic role in AD prevention. In total, 32 compounds were observed to be present at higher levels in decaffeinated coffee. Although our data clearly displayed these chemical differences, they could not differentiate if these were due to decaffeination process or post-decaffeination modification processes employed by the manufacturer.

## Conclusions

In this study, we successfully established GC-TOF-MS as a suitable platform for metabolomics analysis of coffee samples. Our metabolomics approach was demonstrated to be a highly robust tool in discriminant analysis between caffeinated and decaffeinated coffee samples. Discriminant metabolites include several phenolic compounds, organic acids, sugar, fatty acids, and amino acids. All these compounds are biologically relevant and our findings provide important revelations into research of therapeutic effects of coffee against AD. Our data also suggest possible involvement of gut microbial metabolism of compounds present in caffeinated coffee, which could have enhanced its therapeutic potential against AD. This represents an interesting area for future research, which should aim to uncover the links between coffee compositions, gut microbial metabolism, and overall health outcome.

This study has a few limitations, one is that we did not measure absolute concentrations of each discriminant metabolite, which requires an entirely different methodology and it is not in line with our research objectives in this study. Another limitation is that our metabolic coverage is not exhaustive, but to the best of our knowledge, our analytical method offers the widest coverage of coffee metabolites using a single analytical platform with commercially available spectral libraries.

Despite having these limitations, our findings remain significant and novel. This is the first study to evidently demonstrate the wide-ranging chemical differences between caffeinated and decaffeinated coffee, and clearly showed that caffeine is not the only major discriminant metabolite between the two coffee options. Therefore, there exists a need to promptly share this information with others in the field of coffee research.

## Supporting Information

Table S1 **List of 69 metabolites that differentiate caffeinated from decaffeinated coffee samples.**
(DOC)
