# Supplementary material for: Gas Chromatography Time-Of-Flight Mass Spectrometry (GC-TOF-MS)-Based Metabolomics for Comparison of Caffeinated and Decaffeinated Coffee and Its Implications for Alzheimer’s Disease
Source: PLoS One. 2014 Aug 6;9(8):e104621. doi: 10.1371/journal.pone.0104621 (PMC4123969; doi:10.1371/journal.pone.0104621)
Supplement: Table S1 — List of 69 metabolites that differentiate caffeinated from decaffeinated coffee samples. (DOC) [file pone.0104621.s001.doc]

|  |  |  |  | **% metabolite levels relative to caffeinated coffee, mean ± SD** | |
| --- | --- | --- | --- | --- | --- |
| **S/N** | **Metabolite** | **VIP ^a^** | **SI (%)^b^** | **Caffeinated** | **Decaffeinated ^c^** |
| 1 | Caffeine | 1.0873 | 91.0 | 100.00 ± 3.48 | 12.26 ± 0.46 |
| 2 | Fumarate | 1.0883 | 77.6 | 100.00 ± 1.31 | 33.85 ± 0.56 |
| 3 | 4-Hydroxybutyrate | 1.0884 | 91.6 | 100.00 ± 0.96 | 35.50 ± 0.86 |
| 4 | Malonate | 1.0884 | 80.2 | 100.00 ± 0.83 | 35.60 ± 0.97 |
| 5 | 3-Hydroxybenzoate | 1.0869 | 91.0 | 100.00 ± 2.19 | 39.88 ± 1.60 |
| 6 | 2-(2-Hydroxyphenyl)ethanol | 1.0883 | 77.0 | 100.00 ± 1.16 | 41.29 ± 0.67 |
| 7 | 5-Hydroxyvalerate | 1.0865 | 74.2 | 100.00 ± 2.78 | 42.00 ± 0.92 |
| 8 | 2-Deoxy-D-galactose | 1.0459 | 81.8 | 100.00 ± 11.51 | 46.22 ± 4.29 |
| 9 | Deoxyribose 1-phosphate | 1.0865 | 78.9 | 100.00 ± 2.25 | 49.05 ± 1.25 |
| 10 | Arabinose | 1.088 | 73.6 | 100.00 ± 1.18 | 50.50 ± 0.80 |
| 11 | L-2-hydroxyglutarate | 1.0882 | 90.7 | 100.00 ± 0.99 | 52.32 ± 0.56 |
| 12 | 2-Deoxy-erythro-pentonate | 1.0881 | 72.4 | 100.00 ± 0.91 | 54.73 ± 0.78 |
| 13 | 2-methylpipecolate | 1.0867 | 88.6 | 100.00 ± 1.19 | 56.47 ± 1.75 |
| 14 | Uracil | 1.0866 | 86.1 | 100.00 ± 1.69 | 59.20 ± 1.05 |
| 15 | 4,6-dimethoxy-5-(acetyl)pyrimidine | 1.0846 | 81.1 | 100.00 ± 2.53 | 59.77 ± 1.12 |
| 16 | Erythronate | 1.0853 | 84.9 | 100.00 ± 1.84 | 59.98 ± 1.73 |
| 17 | Levoglucosan | 1.0817 | 80.0 | 100.00 ± 3.00 | 62.06 ± 1.63 |
| 18 | 2-Hydroxyaniline | 1.0879 | 80.2 | 100.00 ± 0.70 | 62.07 ± 0.87 |
| 19 | Methylsuccinate | 1.0865 | 91.5 | 100.00 ± 1.62 | 62.25 ± 0.95 |
| 20 | Shikimate | 1.0867 | 77.1 | 100.00 ± 1.69 | 62.59 ± 0.58 |
| 21 | L-rhamnose | 1.0431 | 83.1 | 100.00 ± 7.82 | 63.06 ± 3.89 |
| 22 | Hydroxyisocaproate | 1.0795 | 82.7 | 100.00 ± 3.50 | 64.09 ± 1.28 |
| 23 | 2-Furanpropanoate | 1.0859 | 82.3 | 100.00 ± 1.61 | 68.57 ± 0.75 |
| 24 | D-glucose | 1.0796 | 94.4 | 100.00 ± 2.71 | 69.54 ± 1.57 |
| 25 | 2-Hydroxybutyrate | 1.0692 | 88.4 | 100.00 ± 2.05 | 72.76 ± 3.60 |
| 26 | 4-Hydroxybenzoate | 1.0512 | 88.9 | 100.00 ± 2.26 | 74.95 ± 4.84 |
| 27 | D-galactose | 1.0766 | 95.2 | 100.00 ± 2.50 | 75.11 ± 1.60 |
| 28 | Picolinate | 1.0043 | 82.3 | 100.00 ± 5.25 | 76.46 ± 5.76 |
| 29 | Lactic acid dimer | 1.0637 | 74.8 | 100.00 ± 2.51 | 81.31 ± 2.03 |
| 30 | Benzoate | 1.0599 | 77.5 | 100.00 ± 2.75 | 82.48 ± 1.74 |
| 31 | Succinate | 1.0851 | 94.1 | 100.00 ± 0.72 | 83.13 ± 0.83 |
| 32 | 2-Furoate | 1.0831 | 94.3 | 100.00 ± 1.00 | 83.17 ± 0.92 |
| 33 | D-fructose | 1.0692 | 89.6 | 100.00 ± 2.09 | 83.20 ± 1.47 |
| 34 | 4-Hydroxy-2-pentenoate | 1.0758 | 78.5 | 100.00 ± 1.46 | 83.45 ± 1.41 |
| 35 | 3-Deoxytetronate | 1.0812 | 89.6 | 100.00 ± 0.47 | 85.59 ± 1.27 |
| 36 | 2,3-Dimethyl-3-hydroxyglutarate | 1.0187 | 76.9 | 100.00 ± 2.36 | 91.48 ± 0.96 |
| 37 | Lactate | 1.0264 | 95.5 | 100.00 ± 1.08 | 95.09 ± 0.85 |
| 38 | Parabanate | 1.0644 | 77.0 | 100.00 ± 1.43 | 109.43 ± 0.73 |
| 39 | 3-Deoxy-ribo-hexonate, 1,4-lactone | 1.0124 | 81.6 | 100.00 ± 1.77 | 109.54 ± 2.40 |
| 40 | Protocatechuate | 1.0535 | 80.2 | 100.00 ± 1.79 | 111.12 ± 1.43 |
| 41 | 2-Deoxytetronate | 1.0689 | 81.6 | 100.00 ± 1.04 | 114.08 ± 1.89 |
| 42 | D-ribose | 1.0578 | 87.2 | 100.00 ± 1.82 | 115.76 ± 2.43 |
| 43 | 2,3-Hydroxypropanoate | 1.083 | 90.9 | 100.00 ± 1.82 | 119.78 ± 1.20 |
| 44 | Gentisate | 1.0825 | 79.0 | 100.00 ± 1.41 | 119.90 ± 0.93 |
| 45 | 1-Methyl-2-piperidinemethanol | 1.0804 | 86.5 | 100.00 ± 0.60 | 120.84 ± 1.96 |
| 46 | D-(+)-Fucose | 1.0834 | 84.3 | 100.00 ± 0.76 | 122.60 ± 1.61 |
| 47 | Methylmaleate | 1.0342 | 88.8 | 100.00 ± 3.52 | 125.82 ± 5.73 |
| 48 | Sumiki's acid | 1.0866 | 93.6 | 100.00 ± 0.58 | 129.10 ± 1.30 |
| 49 | Hydroxypropionate | 1.0841 | 90.3 | 100.00 ± 1.56 | 133.39 ± 1.89 |
| 50 | Uridine | 1.0589 | 82.4 | 100.00 ± 3.46 | 136.20 ± 5.90 |
| 51 | Maleate | 1.0752 | 86.6 | 100.00 ± 2.13 | 138.46 ± 4.35 |
| 52 | Trigonelline | 1.0305 | 84.4 | 100.00 ± 8.17 | 141.00 ± 7.45 |
| 53 | Ribitol | 1.0694 | 88.6 | 100.00 ± 3.80 | 141.90 ± 5.05 |
| 54 | D-xylonate 1,4-lactone | 1.0788 | 81.8 | 100.00 ± 2.24 | 150.64 ± 4.96 |
| 55 | Glycine | 1.0842 | 85.9 | 100.00 ± 2.78 | 152.85 ± 2.62 |
| 56 | L-valine | 1.0559 | 89.4 | 100.00 ± 7.80 | 153.73 ± 7.29 |
| 57 | D-glucuronate | 1.0419 | 82.9 | 100.00 ± 8.61 | 155.21 ± 10.05 |
| 58 | L-alanine | 1.062 | 88.4 | 100.00 ± 5.81 | 158.97 ± 8.77 |
| 59 | D-xylopyranose | 1.0663 | 75.1 | 100.00 ± 4.86 | 161.74 ± 8.80 |
| 60 | L-phenylalanine | 1.0685 | 77.4 | 100.00 ± 4.74 | 176.39 ± 10.81 |
| 61 | N-formylglycine | 1.0604 | 79.0 | 100.00 ± 4.90 | 181.39 ± 14.14 |
| 62 | L-proline | 1.0392 | 79.8 | 100.00 ± 6.32 | 184.75 ± 19.95 |
| 63 | Furazan | 1.0686 | 97.8 | 100.00 ± 9.10 | 186.00 ± 9.62 |
| 64 | 2-Deoxy-pentos-3-ulose | 1.0765 | 82.4 | 100.00 ± 1.65 | 188.39 ± 10.42 |
| 65 | Malate | 1.0869 | 92.3 | 100.00 ± 2.86 | 196.25 ± 3.37 |
| 66 | L-aspartate | 1.0612 | 88.3 | 100.00 ± 8.17 | 228.83 ± 21.89 |
| 67 | 2-Ketobutyrate | 1.0885 | 83.9 | 100.00 ± 4.54 | 408.99 ± 2.23 |
| 68 | Guaiacol-alpha-glucoside | 1.0881 | 74.7 | 100.00 ± 3.67 | 688.59 ± 14.56 |
| 69 | Pyruvate | 1.0885 | 81.2 | 100.00 ± 4.87 | 885.53 ± 10.34 |

^a^ Variable importance plot (VIP) values were generated from orthogonal partial least squares discriminant analysis (OPLS-DA) model.

^b^ Similarity index (SI) indicates percentage match between the mass spectrum of identified compound in samples versus library spectrum.

^c^ All 69 metabolites achieved significance (*P* < 0.0007, Bonferroni-adjusted significance level). Means were compared using two-tailed independent t-tests with Welch’s correction.
